# Wild chimpanzees *(Pan troglodytes troglodytes*) exploit tortoises *(Kinixys erosa)* via percussive technology

**DOI:** 10.1038/s41598-019-43301-8

**Published:** 2019-05-23

**Authors:** Simone Pika, Harmonie Klein, Sarah Bunel, Pauline Baas, Erwan Théleste, Tobias Deschner

**Affiliations:** 1 University of Osnabrück, Institute of Cognitive Science, Comparative BioCognition, Artilleriestrasse 34, 49076, Osnabrück, Germany; 2 Max Planck Institute for Evolutionary Anthropology, Department of Primatology, Deutscher Platz 6, 04103, Leipzig, Germany

**Keywords:** Animal behaviour, Biological anthropology

## Abstract

Chimpanzees (*Pan troglodytes*), one of humankinds’ closest living relatives, are known to hunt and consume the meat of various animal taxa. Although some researchers have presented indirect evidence that chimpanzees may also prey on tortoises, until now, direct observations of this behaviour did not exist. Here, we provide systematic descriptions of the first observations of chimpanzee predation on tortoises (*Kinixys erosa*). We made these unprecedented observations on newly habituated chimpanzees (*Pan troglodytes troglodytes*) of the Rekambo community, living in the Loango National Park, Gabon. The behaviour qualified as customary, that is occurring in most or all adult males, involved a distinct smashing technique, and resulted frequently in food sharing with other group members. Our observations shed new light on the hitherto little understood percussive technology of chimpanzees, and expand our current knowledge on chimpanzees’ dietary and predatory repertoires with respect to reptiles. We also report a case of food storage and discuss it in the context of future-oriented cognition. Our findings suggest the need for more nuanced interpretations of chimpanzees’ cognitive skills in combination with an in-depth understanding of their unique socio-ecological niches. They further emphasize the importance of nonhuman primate field observations to inform theories of hominin evolution.

## Introduction

The ability to use and manufacture tools, long thought to be uniquely human, has now been reported in a variety of species of birds (e.g., New Caledonian crows *Corvus moneduloides*)^1^ and mammals (e.g., sea otter *Enhydra lutris*^2^; bottlenose dolphins *Tursiops* sp.^3^). Scholars distinguish between tool-use and proto-tool use, although there is no evidence that proto-tool use is an evolutionary or ontogenetic precursor to tool use^4^. Tool use is defined as “the external employment of an unattached or manipulable attached environmental object to alter more efficiently the form, position, or condition of another object, another organism, or the user itself, when the user holds and directly manipulates the tool, during or prior to use, and is responsible for the proper and effective orientation of the tool”^4,5^ (p. 5) (e.g., sea otter opening the shell of an abalone by pounding it on a stone that is balanced on the otter’s ventrum)^2^. Proto-tools, on the other hand, are functionally analogous to tools but are not held and directly manipulated during or prior to use (e.g., labrid fishes *Cheilinus trilobatus* and *Coris angulata* pound sea urchins on coral or stones to detach the spines and smash the exoskeletons).

Compared to other nonhuman animals, chimpanzees (*Pan troglodytes*) have developed an exceptionally large and varied technological repertoire^6,7^. For instance, they deploy tools made from diverse plant sources to acquire social insects, and utilize spear-like tools to rouse prosimian prey from cavities^7–10^. In addition, they are known to use percussive technology, ranging from pounding open hard-shelled fruits (*Baobab* sp.^11^, *Strychnos* sp.^12^) and termite mounds^13^ against substrates to pounding open nuts (*Coula edulis, Panda oleosa*) using hammers^14,15^. Some researchers have also suggested that chimpanzees employ percussive technology to prey on tortoises^16,17^, but direct evidence was, until now, non-existent. Concerning humans, the predation and consumption of tortoises has already been attributed to early hominins (*Homo erectus*; ≈*1.9 mya*)^18^. Tortoises also represent an important food resource in the diet of present-day hunter-gatherer societies^19,20^.

Here, we provide the first direct observations of tortoise predation in wild chimpanzees (*Pan troglodytes troglodytes*). The behaviour of individuals of the Rekambo community, Loango National Park (Gabon) was observed on a daily basis from July 2016 – May 2018. Tortoise predation events were, however, restricted to the dry season only. The behaviour is frequently shown by the majority of adult males of the Rekambo community, thereby qualifying as *customary*^7^. In addition, we report on a single case of food storage, in which an adult male tucked a half-eaten tortoise in a tree fork and retrieved it the next day to continue feeding.

Our results shed new light on the percussive technology of chimpanzees. They also expand our current knowledge on the relatively broad dietary and predatory repertoires of these ecologically flexible omnivores to reptiles. Furthermore, although chimpanzees are renowned to frequently hunt and consume the meat of a variety of vertebrates (most heavily red colobus *Piliocolobus* spp., as well as black-and-white colobus monkeys *Colobus* spp., red-tailed guenons *Cercopithecus ascanius*, olive baboons *Papio anubis*, red duikers *Cephalophus natalensis*, and bushpigs *Potomochoerus porcus*)^6,21,22^, food storage in primates has so far only been implicated for early hominins^16^.

## Results

**Figure 1:**
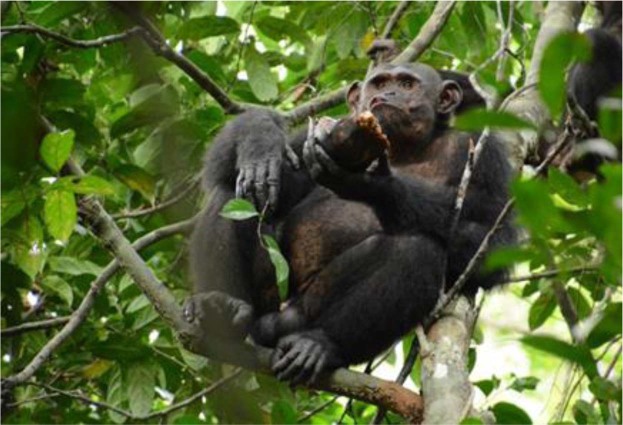
Tortoise predation. Chimpanzee handling and consuming a predated tortoise ©Théleste.

We observed 38 prey events (34 successful and four unsuccessful ones) on hinge-back tortoises (*Kinixys erosa*) by ten different chimpanzees (seven adult males, one adult female, one adolescent male, one adolescent female) of the Rekambo community in the Loango National Park, Gabon (see Table 1, Figure 1 and video files in the Supplementary Material).

**Table 1:** Tortoise predation events.

Individual	Sex	Age class	First ownership	Discovery	Smashing success/N of total trials^a^	Transportation	Food sharing^b^	Meat access^c^
Pandi	male	adult	4	14	20/20	4	13	2
Littlegrey	male	adult	1	3	4/4	0	2	5
Onoumbou	male	adult	1	1	4/4	1	3	4
Louis	male	adult	1	1	3/3	1	3	4
Freddy	male	adult	0	3	1/2	1^d^	0	7
Thea	male	adult	1	2	1/2	0	1	2
Chinois	male	adult	1	0	1/1	0	1	6
Orian	male	adolescent	0	0	0	0	0	5
Ngonde	male	adolescent	0	0	0	0	0	1
Suzee	female	adult	0	1	0/1	0	0	1
Roxy	female	adult	0	0	0	0	0	1
Joy	female	adult	0	0	0	0	0	1
Gia	female	adolescent	2	1	0/2	0	0	1
Unknown^e^	male	adolescent	0	1	0/1	0	0	1
Unknown^e^	female	adolescent	0	0	0	0	0	1
**Total**			**11**	**27**	**34/41**	**7**	**23**	**42**

Tortoise predation events as a function of individual, sex and age class, as well as total numbers for discoveries, smashing trials and success, transportation of tortoise, food sharing and meat access for the data collection period from January 2016 to May 2018. The category of first ownership depicts those events where we did not observe the discovery of the tortoise. It refers to the first individual observed handling the tortoise.

^a^The category “successful smashing” includes smashing cases irrespectively of discovering the tortoise.

^b^In all those cases when we observed begging, meat was shared by the targeted individual. The absence of meat sharing was due to: (1) the meat owner being alone, and (2) conspecifics failing to approach the meat owner.

^c^Number of times when the targeted individual had access to meat during the entire period of sharing. The numbers refer to a single count per event.

^d^Transportation of the tortoise without the chimpanzee subsequently smashing the plastron.

^e^Individual/s that was/were present during the tortoise predation event but could not be identified.

All predation events were observed in periods of high fruit availability and in the dry season^23^ (July 2016, May to October 2017, May to June 2018). We were able to observe 13 prey events from the discovery of the tortoise until meat consumption. The most frequent behavioural sequence observed consisted of: (1) discovering and capturing the prey, (2) smashing the plastron with one hand repeatedly against a wooden anvil (trunk: N = 9; for other anvils used see Table S1), and (3) climbing into a tree to consume the meat (N = 9; see detailed information in Table S1). The smashing mainly occurred in a horizontal manner against the anvil (N = 22). In six cases, the chimpanzee first transported the tortoise between 10–50 m on the ground, for a duration of one to eleven minutes, before smashing it. For ten of the 13 events, the chimpanzee first smashed the tortoise against an anvil while on the ground and then smashed it again against another anvil after having climbed up a tree (against a branch: N = 5; or the trunk: N = 5). In 29 cases, the chimpanzee, who was first seen handling the tortoise was also successful in smashing it open. In six cases, the individual, who had discovered the tortoise, tried to smash the plastron but did not succeed in opening it. On three of these occasions, the chimpanzee then abandoned the prey. In two of these cases, an older individual then approached the tortoise and succeeding in smashing it open (see Table 1). In two cases, there was an intermediate possessor of the prey, both times a female, before the tortoise was retrieved and successfully opened. Food sharing between meat owners and other members of the party occurred in 23 of all observed events. Three individuals, that had discovered a tortoise but had not been successful in opening it (N = 5), received meat from the successful openers (including two events with an unidentified female). In one case, we observed a tortoise predation event by the alpha male, who was on his own. After consuming approximately half of the tortoise meat while sitting in a tree, he clamped the remaining parts into a fork of the tree, climbed down, and travelled for approximately 100 meters to build a nest in a nearby tree. He slept overnight and left his nest 13 hours later to retrieve the remaining parts of the tortoise the next morning. He then continued to eat them (for further details see Supplementary Material).

## Discussion

Overall, we found that tortoise predation is a customary^7^ behaviour in the Rekambo community, regularly done by all adult males. It most frequently consisted of a distinct sequence of behaviours involving the discovery of the prey, transportation of the prey to a suitable anvil, smashing of the plastron, and feeding on the meat. The majority of tortoises were successfully cracked open by adult male chimpanzees. In the two cases where adolescent chimpanzees attempted to smash open a tortoise, they were unsuccessful. Similar to nut cracking in chimpanzees^14^ — a percussive technology which is only mastered at the age of approximately nine to ten years^15^ — the acquisition of a successful tortoise smashing technique may rely on a certain amount of strength. In addition, it may also involve a relatively long period of time to learn, practise and refine^14,15^.

The majority of tortoise predation events observed resulted in peaceful, cooperative food sharing^24^ with other individuals of the party (i.e., lacking aggressive interactions and appeasement gestures). Unsuccessful tortoise smashers also received a share of the meat in nearly all observed instances. These observations are in line with observations of meat sharing events at other long-term field sites such as Ngogo, Uganda^25,26^ and Taï, Ivory Coast^8^, where individuals share actively with conspecifics (however see)^27^.

So far, tortoise predation has been reported in a variety of animal taxa, including different bird species (e.g., Verreaux’s eagles *Aquila verreau*^28^; common ravens *Corvus corax*^29^; kelp gulls *Larus dominicanus*^30^), several carnivore species (e.g., raccoons *Procyon lotor*^31^; cougars *Puma concolor*, bobcats *Lynx rufus*, coyotes *Canis latrans*^32^; black bears *Ursus americanus*^33^; honey badgers *Mellivora capensis*^34^), and two nonhuman primate species (i.e., mandrills *Mandrillus sphinx*^35^; chacma baboons *Papio cynocephalus ursinus*^36^). The techniques used to gain access to the meat and organs include breaking through the tortoise’s integuments^31,37^, head, feet or soft parts of the plastron using canines^35,36^ or beaks^29,30^. Some species have been reported to use their hands^34^ or beaks^29^ to tear the plastron, or the head and/or legs to gain access to the entrails. In addition, some species of birds (e.g., white-necked ravens *Corvus albicollis;* Egyptian vultures *Neophron percnopterus*)^38^ lift tortoises in the air and drop them onto rocks. This behaviour qualifies as percussive proto-tool use, with the hard substrate (i.e., the rock) not being held and directly manipulated during or prior to use^4^. However, the use of a percussive technology to gain access to tortoises’ interior has never previously been described in a nonhuman primate species.

Overall, our findings of an unreported percussive technique add another intriguing facet to the variability of techniques developed in the animal kingdom to prey upon tortoises. The discovery broadens the range of food items accessed by chimpanzees using percussive technology such as nuts (*Coula edulis, Panda oleosa*), hard-shelled fruits (*Baobab* sp.^11^, *Strychnos* sp.^12^, *Treculia africana*^39^), termite mounds^13^, skulls of monkeys^24^, and possibly snails (*Achatina achatina*)^13^. They thus add new support for the hypothesis of Marchant and McGrew^11^ postulating that percussive technology originated in ancestral hominoids with hard-shelled fruits being smashed against tree trunks and boughs. Even more importantly, our findings shed new light on chimpanzees’ feeding ecology and underlying flexibility. So far, our understanding of their dietary and predation repertoires had rested on a broad range of taxa including birds, insects, mammals, and possibly gastropods^6,13,22^. For instance, they have been observed to consume at least 25 different species of mammals ranging in size from small rodents (<1 kg) to juvenile bush pigs (>20 kg)^6,22^. Our observations now expand this extensive list to include another food resource: reptiles.

There are several alternative hypotheses as to why tortoise predation has so far only been observed in the Rekambo community during the dry season. First, it may be possible that our discovery of tortoise predation is the result of the increased number of observations following chimpanzee habituation. If this is true, we predicted to observe predation events more and more often with increasing habituation level, independent of any seasonal pattern. This, however, was not the case (see Table S1 in the Supplementary Material). Second, activity patterns of hinge-back tortoises in Cameroon^40^ and Nigeria^41^ are characterized by activity peaks during the dry season (May to October) and prolonged resting periods during the rainy season (November to April). Assuming that hinge-back tortoises in Gabon show similar activity patterns, chimpanzees should then be more likely to discover them as they are moving around (during the dry season). Third, chimpanzees may more easily discover hinge-back tortoises in the dry season due to the noise created by their movement through the dry leaf litter. Fourth, the diversity and access to crucial feeding resources may differ between the rainy and the dry season, resulting in chimpanzees shifting their ranging patterns, use of habitat and exploitation of food resources. Future research will hopefully enable us to distinguish among these alternative hypotheses.

Why tortoise predation has never directly been observed at any other long-term chimpanzee field site remains a puzzling question. One explanation may be that the ecological niche of chimpanzees only rarely overlaps with that of hinge-back tortoises. Hinge-back tortoises show a relatively rich ecological diversity and are found in lowland evergreen forest, marshy areas, and gallery forests growing along rivers and streams^42–44^. For instance, Vonesh^44^ reported that hinge-back tortoises are widely distributed throughout Uganda, but seem to be absent from intact forest areas with relatively high densities of chimpanzees such as the Kibale National Park. Another explanation may be that individuals of chimpanzee communities which share their habitats with tortoises — such as for instance in the Taï National Park, Ivory Coast — consume sufficient amounts of other meat resources rendering tortoise predation unnecessary. Alternatively, we may have discovered another cultural behaviour^7^: Tortoise predation via percussive technology may have been invented by a single individual and was then transmitted within the population via social learning.

We observed that in some cases individuals transported the tortoises for several minutes, and over considerable distances, before trying to crack them open on suitable anvils. In one case, as described above, the alpha male positioned a half-eaten tortoise in a nearby tree fork in the late afternoon and retrieved it only the next day. The transportation of the meat resource represents an attempt to satisfy the chimpanzee’s own hunger state, thereby implying planning for a current physiological need^45,46^. This cognitive ability also characterizes many other tool-using behaviours of chimpanzees where tools are transported to out-of-sight locations^6,8,47^.

There are at least two possible explanations for the incident in which the alpha male placed a half-eaten tortoise in a tree fork and retrieved it for final consumption only the next day. First, the chimpanzee, being satiated, may have simply left the dead prey in the tree fork, climbed down and built a nest in a nearby tree. The next day, when awakening with hunger, he remembered the meat resource and climbed up into the tree to retrieve it. If this explanation is true, it strengthens recent findings showing that similar to many food-hoarding birds, carnivores and rodents^48–50^, chimpanzees rely on sophisticated memory skills to remember and retrieve food items many hours later^51^. However, this explanation does not account for the chimpanzee placing the dead tortoise in a tree fork instead of merely dropping it to the ground after finishing the meal. Hence, a second explanation is that the chimpanzee may have intentionally (see for definition)^52^ deposited the half-eaten tortoise into the tree fork involving future-oriented cognition: he anticipated a future need, that was different from his current state. Planning for a future mental state is cognitively demanding because it imposes a long delay between performing an action and getting rewarded for it^46^. Although future-oriented cognition is still considered by some scholars to be uniquely human^53^, evidence for it — based on experimentally induced behaviours in captive species — has been found in pigeons (*Columba livia*)^54^, rats (*Rattus norvegicus domesticus*)^55^, corvids^46,56^ and great apes^57,58^. The only systematic evidence of spontaneous future-oriented cognition in a species living in its natural environment stems from Western chimpanzees (*Pan troglodytes verus*)^59^. In this study, adult females seemed to flexibly plan when and where they will have breakfast the following morning based on the assessment of multiple factors which influences where they choose to build their night nests^59^.

Although we only observed a single case of possible food storage, tortoise predation may be a useful candidate to investigate evolutionary precursors to human central place foraging and central place provisioning^60,61^. For instance, McGrew^16^ suggested that chimpanzees lack distinct behaviours that may have been important for central place foraging and central place provisioning, thereby paving the way to hominization. For example, tools for obtaining vertebrate prey and a means of collecting and transporting gathered food for exchange. However, a hinge-back tortoise discovered at the end of the day may represent a food surplus embedded in its own natural container which can be readily transported^62^. Theories of hominid evolution could thus immensely benefit by interdisciplinary exchanges and collaborations between the fields of Anthropology, Palaeoarchaeology and Primatology^63^.

Our results strongly emphasize the need for more nuanced interpretations of chimpanzees’ cognitive skills in combination with an in-depth understanding of their unique socio-ecological niches. In the particular case described here of possible food storage observed at Loango, the unique combination of a very specific prey species (which, due to its encapsulation may be inaccessible to other predators) and the discovery of this tortoise by a solitary male (without the risk of the prey being stolen by group members) may have created a unique opportunity: to plan for a future need by avoiding discovery of the prey by other chimpanzees and non-chimpanzee predators during the night. Reliable insights into the purpose cognitive abilities serve^64^ can thus only be gained by unravelling specific socio-ecological factors favouring their emergence − a task demanding careful, knowledgeable observations of species living in their natural environments.

In sum, our findings strongly emphasize the versatility and diversity of chimpanzees’ percussive technology and provide further support for their exceptionally large and flexible cognitive tool kits. One of our observations — possible food storage of a tortoise in a tree fork overnight — may even question the hypothesis that future-oriented cognition is a uniquely human attribute^49^. Cognitive precursors for future-orientated planning may have thus evolved in great apes before the evolutionary split between ancestral hominins and *Pan*^57^. Hence, future investigations into the peculiar behaviour of tortoise predation may provide useful insight into the origins of ancestral hominid percussive technology^11^ and possible precursors to central place foraging and central place provisioning^60,61^. The transition from ancestral ape through chimpanzee-like proto-hominin to emergent *Homo* may thus be more readily imaginable^16^.

## Methods

We observed the behaviour of individuals of the newly habituated Rekambo chimpanzee community in the Loango National Park, Gabon (2°04′S and 9°33′E). The site is ecologically very distinct from other long-term chimpanzee sites, consisting of a mosaic of different habitat types varying from marine, coastal lagoons, mangrove swamps, coastal forest, secondary and primary forest to open savannah. During the study period, the mean annual rainfall was 3522 mm and mean daily temperatures ranged between 22.3 and 27.9 °C. The long rainy season at Loango lasts from October to April with a small dry season in December. The long dry season begins in May and lasts until September. More details of the ecological conditions at this field site have been provided elsewhere^23^. The habituation of the Rekambo community began in 2005, with the majority of adult males having been habituated to human presence by 2017. Currently, approximately 20 individuals (eight adult males, four adolescent males, at least five females, one juvenile male and two juvenile females) are well habituated, allowing for systematic behavioural data collection including the collection of high-quality video footage. The data collection period for this study was July 2016 to May 2018, with most observations made during May 2017 to October 2017. The total observation time was approximately 5018 hours over 566 non-consecutive days. Observations were made opportunistically between 07:00 and 18:00 (dawn to dusk; sampling method: all occurrences of some behaviours and event sampling)^65^. Data collection was performed via customized CyberTracker software (Cyber Tracker 3.441) on water-resistant smart phones (Samsung Galaxy Xcover 3).

### Tortoise species and data collection

Forest hinge-back tortoises (Family Testudinidae) are medium- to large-sized reptiles with a carapace of approximately 400 mm length (weighing about 600–1500 g)^42,43^. Males are larger than females. Hinge-back tortoises inhabit lowland evergreen forest, marshy areas, and gallery forests growing along rivers and streams with a considerable distribution over the continuous Guinea–Congo rainforest region in West and Central Africa. They are one of the most common tortoise species in Gabon, with an omnivorous diet mainly consisting of mushrooms^42,43^.

Specific data collection on tortoise predation started whenever a chimpanzee was found in the possession of a tortoise and/or seemed to be approaching one. In some cases, we found the chimpanzees already transporting a tortoise (N = 3; for details see Table S1). In other cases, we heard the sounds of pounding prior to seeing and approaching the chimpanzee (N = 8). However, in the majority of tortoise predation events, we were able to observe the whole sequence including discovery, smashing, meat consumption, and/or abandoning of the tortoise (see Table S1). We provide information on two tortoise predation events with relatively little detailed observations because they had been made by one of our field assistants before the development of a systematic data collection protocol. Tortoise predation was reported by each observer on an *ad libitum* basis^65^, and included descriptions of every behaviour observed such as the chimpanzee (1) discovering the prey, (2) smashing the prey, (3) anvil/s used, (4) smashing success, (5) transportation, (6) opening of the plastron, (7) meat consumption, as well as (8) party size, and (9) occurrence of food sharing. In addition, whenever possible we recorded the predation events via digital cameras (Canon Legria HF M41) and/or smart phones. The identification of the tortoise species was achieved by ET and TD comparing carapaces with published descriptions on tortoises in National Parks of Gabon^42,43,66^.

#### Ethics statement

In an effort to avoid influencing the natural behaviour of the individuals and groups, our study was purely observational and non-invasive with audio and video recordings taken from a minimum distance of seven meters. The research adhered to the legal requirements of Gabon and followed the recommendations of the ‘Animals (Scientific Procedures) Act 1986’, as published by the government of the United Kingdom, and the principles of “Ethical Treatment of Non-Human Primates” as stated by the American Society of Primatologists. Permission to conduct research at the Loango National Park was granted by the Agence Nationale des Parcs Nationaux, and the Centre National de la Recherche Scientifique et Technique of Gabon, Libreville, Gabon.

## Supplementary information

Video 1: Tortoise smashing, opening and meat consumption

Video 2: Tortoise meat consumption

Video 3: Meat sharing and consumption after tortoise predation
